# Effect of Zinc Acetate Concentration on Optimization of Photocatalytic Activity of *p-*Co_3_O_4_/*n*-ZnO Heterostructures

**DOI:** 10.1186/s11671-018-2604-4

**Published:** 2018-07-05

**Authors:** Hongyan Xu, Mingliang Shi, Caiqin Liang, Siyan Wang, Chengkai Xia, Chenyang Xue, Zhenyin Hai, Serge Zhuiykov

**Affiliations:** 1 School of Materials Science and Engineering, North University of China, Taiyuan, 030051 People’s Republic of China; 2 Key Laboratory of Instrumentation Science and Dynamic Measurement of Ministry of Education, North University of China, Taiyuan, 030051 People’s Republic of China; 3 Department of Applied Analytical and Physical Chemistry, Ghent University Global Campus, 119 Songdomunhwa-ro, Yeonsu-gu, Incheon, 21985 South Korea

**Keywords:** Co_3_O_4_/ZnO, Heterostructures, Photocatalysis, Hydrothermal decomposition

## Abstract

**Abstract:**

In this work, *p*-Co_3_O_4_/*n*-ZnO heterostructures were fabricated on Ni substrate by hydrothermal-decomposition method using cobaltous nitrate hexahydrate (Co(NO_3_)_2_·6H_2_O) and zinc acetate dihydrate (Zn(CH_3_COO)_2_·2H_2_O) as precursors with zinc acetate concentration varying from 5.0 to 55.0 mM. Structure and morphology of the developed samples were characterized by X-ray diffraction (XRD), Raman spectroscopy, and scanning electron microscopy (SEM). Effect of zinc acetate concentration on the photocatalytic activity of *p*-Co_3_O_4_/*n*-ZnO heterostructures was investigated by degradation of methyl orange (MO) under the UV light irradiation. The fabricated *p*-Co_3_O_4_/*n*-ZnO heterostructures exhibited higher photocatalytic activity than pure Co_3_O_4_ particles. In order to obtain the maximum photocatalytic activity, zinc acetate concentration was optimized. Specifically, at 35 mM of zinc acetate, the *p*-Co_3_O_4_/*n*-ZnO showed the highest photocatalytic activity with the degradation efficiency of MO reaching 89.38% after 72 h irradiation. The improvement of photocatalytic performance of *p*-Co_3_O_4_/*n*-ZnO heterostructures is due to the increased concentration of photo-generated holes on Co_3_O_4_ surface and the higher surface-to-volume ratio in the hierarchical structure formed by nano-lamellas.

**Graphical abstract:**

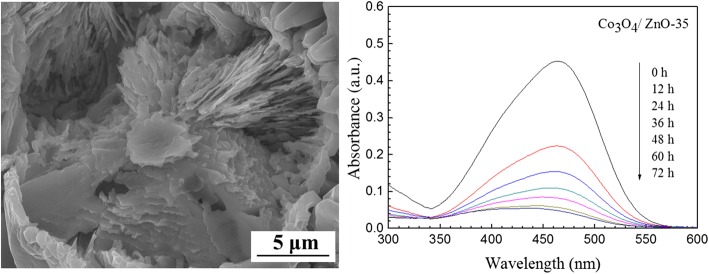

**Electronic supplementary material:**

The online version of this article (10.1186/s11671-018-2604-4) contains supplementary material, which is available to authorized users.

## Background

The rapid development of various industries at the beginning of the twenty-first century has been leading to the fast growing of wastewater at the speed which never been observed in the past. The consequent deterioration of the water quality has been greatly affecting the health of aquatic ecosystems and vast majority of people living in such ecosystems. Hence, the effective water treatment has become one of the major global concerns for the time being [1]. Several modern technologies including physical, chemical, and bio-chemical methods have been developed for the efficient water treatment [2, 3]. Among them, the photocatalysis process has recently gained great attention due to the superior properties of the developed semiconductor catalysts, which have been utilized for the efficient decomposition of various organic pollutants into the smaller and less harmful substances such as CO_2_, H_2_O, and organic short-chain acids [3–8]. Specifically, various micro- and nano-structured semiconductors, such as TiO_2_, MnO_2_, SnO_2_, WO_3_, Fe_2_O_3_, Co_3_O_4_, and ZnO, and different range of their heterojunctions are utilized as functional photocatalysts for the water treatment [9–28]. It is obvious that different photocatalysts have their own benefits and drawbacks. For example, TiO_2_ is so far the most widely employed photocatalyst effective against a wide range of microorganisms co-existing in water. However, it can only absorb UV light for its wide bandgap [12, 13]. On the contrary, ZnO is low cost and nontoxic, but possesses rapid recombination of photo-induced electron-hole pairs [29]. Fe_2_O_3_ has short hole diffusion length (2–4 nm), poor conductivity, and charge recombination [30].

Generally speaking, photocatalysis is based on the reaction between adsorbed molecules (oxygen, surface hydroxyls groups) or water and photo-generated electron/hole pairs excited by the photon with equal or higher energy than the bandgap of semiconductor. However, the electron/hole recombination is blamed for the low quantum yields, which is still a big obstacle for the photocatalytic activity improvement. In order to overcome this obstacle, the development of efficient *p-n* heterojunctions has been proposed and attempted with the different levels of success during last few years. For instance, it was found that the fabricated *p-n* heterojunctions could effectively reduce the recombination rate of the photo-generated electron/hole pairs, which subsequently enhanced the overall photocatalytic activity [31, 32]. Thus, the combination of *p-* and *n-*type semiconductor oxides has paved the way for further development of the *p-n* heterojunctions and optimization of their photocatalytic activity [33].

As an intrinsic *p*-type semiconductor, cobalt oxide (Co_3_O_4_) has been used in the different photocatalytic applications owing to its chemical stability, nontoxicity, low cost, environmental friendliness, etc. [34]. It was reported that Co_3_O_4_ with specific band structure can adsorb oxygen much more efficiently compared to the other *p*-type oxide semiconductors [35]. Other strategies, i.e., doping and heterojunctions, have been developing recently in order to improve the photocatalytic efficiency and properties of the doped Co_3_O_4_-based catalysts such as Co_3_O_4_/Bi_2_WO_6_ [36], Co_3_O_4_/TiO_2_ [37], and Bi_2_O_3_/Co_3_O_4_ [38] have been reported. On the other hand, ZnO (wide bandgap *n*-type semiconductor) has also been intensively studied as one of the best photocatalytic materials due to its high photochemical activity, nontoxic nature, and relatively low cost. Moreover, it was reported that its photocatalytic activity could also be enhanced significantly by modifying its textural characteristics [39]. Therefore, the combination of *p*-type Co_3_O_4_ and *n*-type ZnO represents the right approach for successful development of *p-n* heterojunctions as these heterojunctions can provide built-up inner electric field at the *p-n* interface that can subsequently enhance the overall photocatalytic activity of fabricated composite material. In fact, several different approaches and synthesizing methods for fabrication of these *p*-Co_3_O_4_/*n*-ZnO heterojunctions have recently been reported with reasonable performances [39–41]. However, the optimization of photocatalytic activity of fabricated *p*-*n* heterostructure, which could be linked to the specific micro- or nano-structural variations, to the best of our knowledge, has rarely been addressed.

In this work, *p*-Co_3_O_4_/*n*-ZnO heterostructures were fabricated by hydrothermal decomposition method using cobaltous nitrate hexahydrate (Co(NO_3_)_2_·6H_2_O) and zinc acetate dihydrate (Zn(CH_3_COO)_2_·2H_2_O) as precursors. Their photocatalytic performance was investigated by taking methyl orange (MO) as an example under the UV light irradiation. The developed *p*-Co_3_O_4_/*n*-ZnO heterostructures showed enhancement of the photocatalytic activity in degradation of MO compared to the single Co_3_O_4_ component, as they facilitated more photocatalytic sites and accelerated the surface electron transfer rate due to their much higher surface-to-volume ratio. In addition, the effect of zinc acetate concentration on the photocatalytic activity of *p*-Co_3_O_4_/*n*-ZnO heterostructures was comprehensively investigated and their photocatalytic activity was optimized.

## Results and Discussion

### Characterization of Heterostructures

**Fig. 1:**
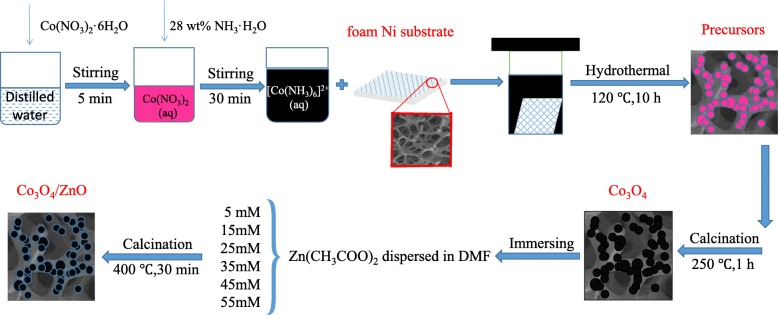
Schematic representation of the fabrication process of *p*-Co_3_O_4_/*n*-ZnO heterostructures

**Fig. 2:**
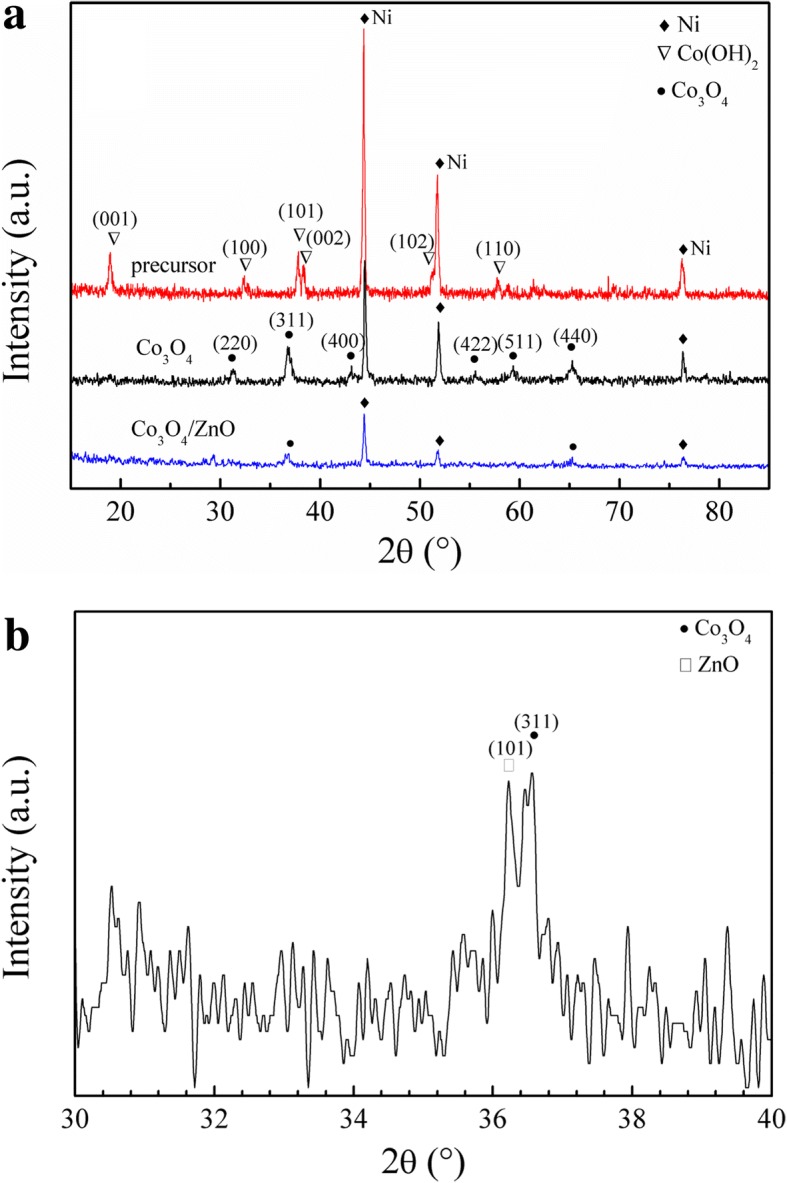
**a** XRD patterns of the precursor, as-prepared pure Co_3_O_4_ and Co_3_O_4_/ZnO-35 heterostructure. **b** Partially enlarged view of the diffraction peaks of Co_3_O_4_/ZnO-35

Figure 1 schematically illustrates the fabrication process of *p*-Co_3_O_4_/*n*-ZnO heterostructures on the Ni substrate. Figure 2 depicts the XRD patterns of the precursor, as-fabricated Co_3_O_4_ and Co_3_O_4_/ZnO-35 heterostructures. The diffraction peaks located at 2*θ* of 44.44°, 51.77°, and 76.31° attributed to (111), (200), and (220) planes, respectively, of Ni (JCPDS card no. 65-2865) observed in all samples [42]. It was found that all identical peaks of the precursor (Fig. 2a) match perfectly to the hexagonal phase of Co(OH)_2_ (JCPDS card no. 30-0443) [43]. The other diffraction peaks at 2*θ* = 19.06°, 32.47°, 37.92°, 38.66°, 51.36°, and 57.91° corresponded to the (001), (100), (101), (002), (102), and (110) planes of Co(OH)_2_, respectively. In the Co_3_O_4_ XRD pattern, the identical Co(OH)_2_ peaks disappeared and new peaks emerged at 31.27°, 36.85°, 44.81°, 55.66°, 59.35°, and 65.23° were attributed and indexed to the crystal planes (220), (311), (400), (422), (511), and (440) of the cubic spinel phase of Co_3_O_4_ (JCPDS card no. 43-1003), respectively [44]. These measurements indicated that pure Co_3_O_4_ is derived from the Co(OH)_2_ after 2 h heating at 250 °C.

XRD pattern of the developed *p*-Co_3_O_4_/*n*-ZnO heterostructure shows that the intensities of diffraction peaks for both Ni substrate and Co_3_O_4_ decreased, which could be caused by the new substance loaded on the surface. In addition, a double-peak can be observed at 2*θ* = 36.5°. Figure 2b displays the partially enlarged pattern of Co_3_O_4_/ZnO, in which (101) peak of ZnO and (311) peak of Co_3_O_4_ are clearly separated. This fact unambiguously confirmed the successful synthesis of the *p*-Co_3_O_4_/*n*-ZnO heterostructures. Moreover, no diffraction impurity peak was detected, which also indicated that the synthesized heterostructures are only made of Co_3_O_4_ and ZnO.

**Fig. 3:**
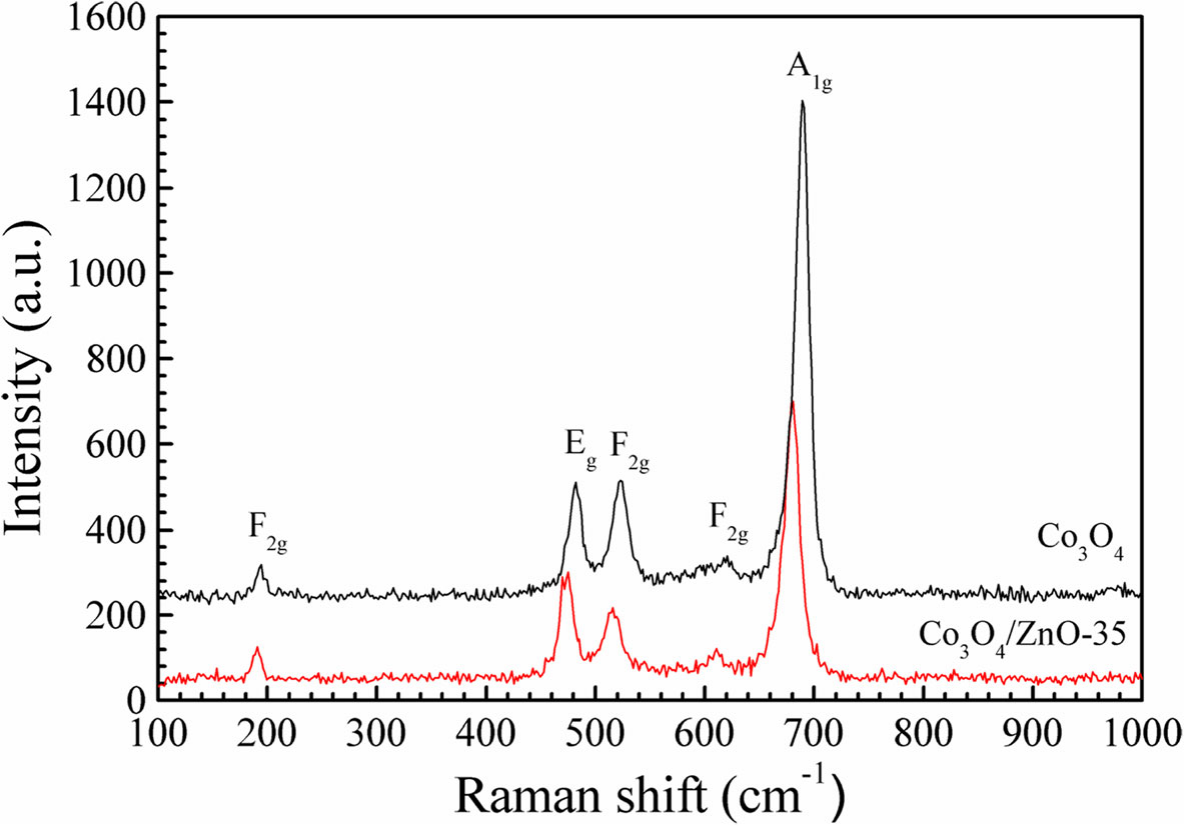
Raman spectra of pure Co_3_O_4_ and Co_3_O_4_/ZnO-35 heterostructure

Figure 3 shows typical Raman spectra of the pure Co_3_O_4_ and the fabricated *p*-Co_3_O_4_/*n*-ZnO heterostructure taken at the room temperature. In these Raman spectra, five different Raman active modes A_1g_ + 3F_2g_ + E_g_ of the Co_3_O_4_ could be observed. It is well known that Co_3_O_4_ has a spinel structure Co^2+^(Co^3+^)_2_O^2−^ _4_ with Co^2+^ and Co^3+^ positioned at tetrahedral and octahedral sites, respectively [45]. A_1g_ mode is a characteristic of the octahedral sites, and the E_g_ and F_2g_ modes are related to the combined vibrations of tetrahedral site and octahedral oxygen motions [46]. Even though, there is no obvious ZnO modes appeared in Co_3_O_4_/ZnO composite, a clear red-shift and broadening features of Co_3_O_4_ Raman modes presented in the spectrum of Co_3_O_4_/ZnO heterostructure. The most intense peak A_1g_ varies from 688.9 cm^− 1^ in pure Co_3_O_4_ to 679.7 cm^− 1^ in Co_3_O_4_/ZnO heterostructure, and its full-width at half maximum (FWHM) changes from 14.61 cm^− 1^ in pure Co_3_O_4_ to 16.02 cm^− 1^ in Co_3_O_4_/ZnO heterostructure. These variations are attributed to the coupling between Co_3_O_4_ and ZnO and also indicated the successful development of Co_3_O_4_/ZnO heterojunction. The same phenomena have been observed in Raman spectra of graphene covered on Ag nanoparticles [47].

**Fig. 4:**
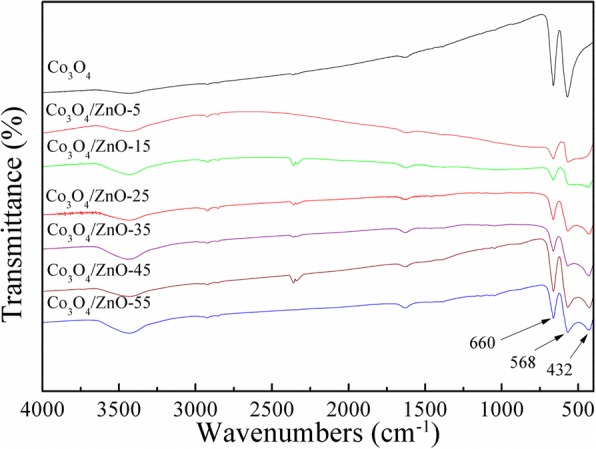
FTIR spectra of synthesized Co_3_O_4_ and Co_3_O_4_/ZnO heterostructures

The FTIR spectra of pure Co_3_O_4_ and Co_3_O_4_/ZnO heterostructures are presented in Fig. 4. The peaks centered at about 3452 and 1634 cm^− 1^ are attributed to the O–H stretching and bending modes of the hydrated oxide surface and the adsorbed water [48, 49]. The IR absorption peaks at about 660 and 568 cm^− 1^ confirm the formation of the phase of spinel Co_3_O_4_ [50]. Compared with the FTIR spectrum of Co_3_O_4_ nanoparticle, new peak at 432 cm^− 1^ appears in all Co_3_O_4_/ZnO FTIR spectra, which is attributed to the existence of ZnO [51]. In addition, the characteristic peak of ZnO at 432 cm^− 1^ becomes sharper with the increasing concentration of zinc source, which confirms the coexistence of ZnO and Co_3_O_4_ and verifies the successful synthesis of ZnO on the Co_3_O_4_ nanoparticles.

**Fig. 5:**
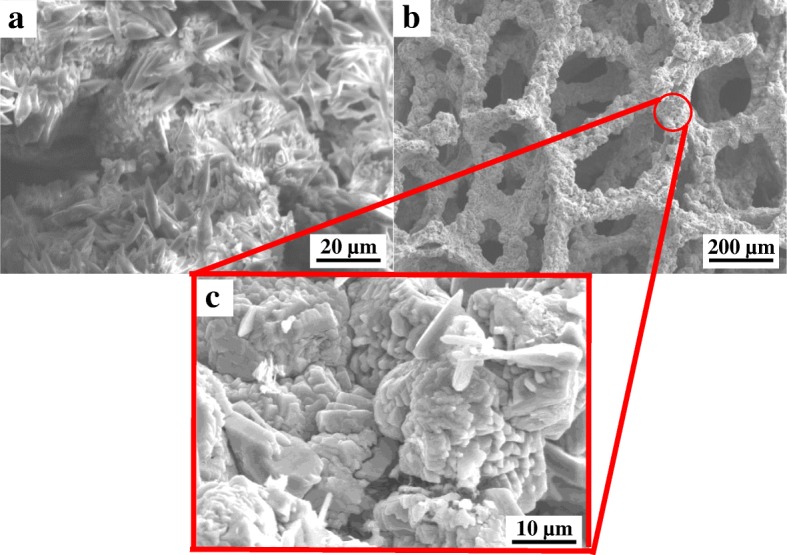
SEM images of **a** Co(OH)_2_ precursor and pure Co_3_O_4_ in **b** low magnification and **c** high magnification

The surface morphology of the precursor and as-prepared pure Co_3_O_4_ are presented in Fig. 5. From the low-magnification SEM image of the Co(OH)_2_ precursor, it is clearly visible that the flower-like layers of precursor, consisting of many sunflower-seed-like petals, have grown uniformly on the surface of the Ni substrate (Fig. 5a). The “petal” size was approximately 10 μm in length, and the whole surface of the porous Ni substrate was covered by Co(OH)_2_ precursor. Furthermore, low-magnification SEM image (Fig. 5b) depicts that the synthetized Co_3_O_4_ crystals are also uniformly and densely covered the porous Ni substrate. High-magnification SEM image (Fig. 5c) shows highly dense structure with lots of “sunflower-seed like” Co_3_O_4_ crystal stacked together to form Co_3_O_4_ spheres. A single sphere size was approximately ~ 20 μm in length. The “sunflower-seed-like” crystals indicated the morphological hereditability of the Co_3_O_4_ from its precursor.

**Fig. 6:**
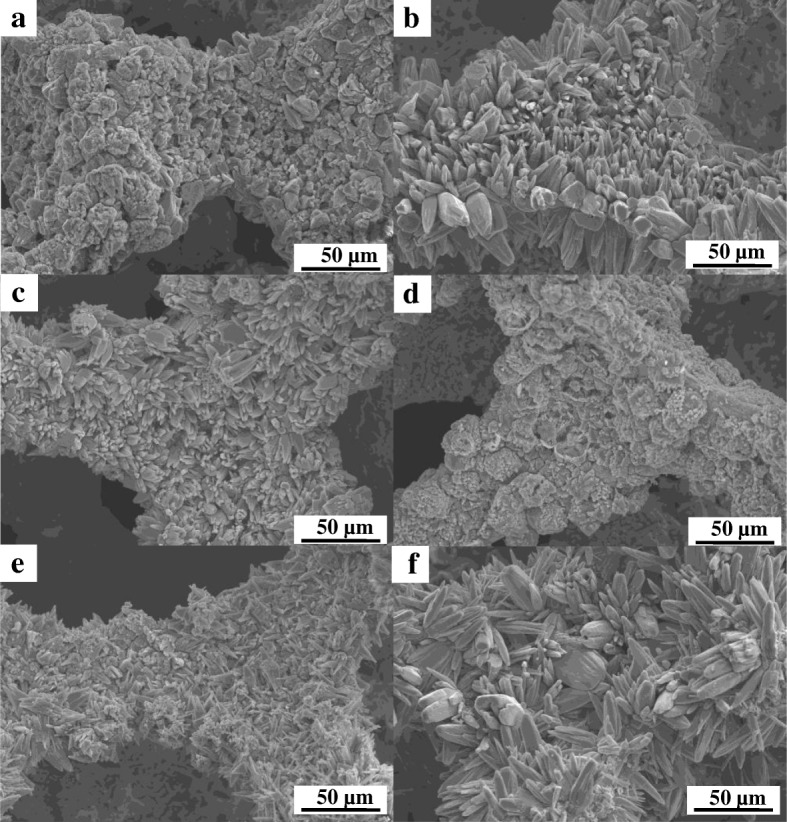
SEM images of *p*-Co_3_O_4_/*n*-ZnO heterostructures grown on the surface of Ni substrate: **a** Co_3_O_4_/ZnO-5, **b** Co_3_O_4_/ZnO-15, **c** Co_3_O_4_/ZnO-25, **d** Co_3_O_4_/ZnO-35, **e** Co_3_O_4_/ZnO-45, and **f** Co_3_O_4_/ZnO-55, respectively

**Fig. 7:**
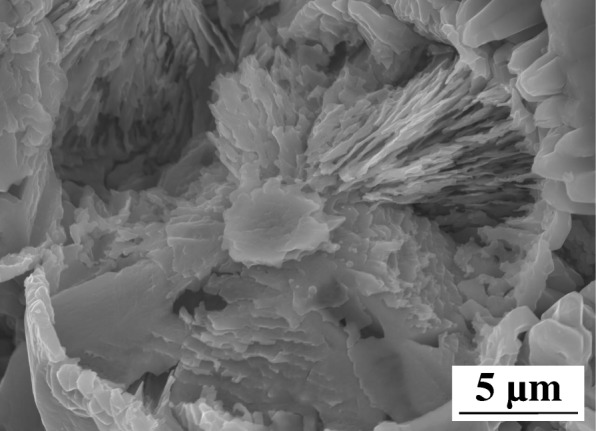
SEM image of the internal structure of Co_3_O_4_/ZnO-35

The morphologies of Co_3_O_4_/ZnO heterostructures, which were fabricated with the different zinc acetate concentrations, were also investigated by SEM, and the main results are summarized in Fig. 6. It is clear evidenced from this figure that the changes in zinc acetate concentration during preparation of Co_3_O_4_/ZnO heterostructures play the crucial role in the development of the morphology variations. For example, the morphology of Co_3_O_4_/ZnO-5 (Fig. 6a) is very similar to the morphology of pure Co_3_O_4_ (Fig. 5c), as the concentration of zinc acetate is low. However, as the concentration of zinc acetate increased from 5.0 to 25.0 mM, the crystallization of sunflower-seed-like small crystals intensified as presented in Fig. 6b, c. What is also interesting is that the size of the sunflower-seed-like crystals appeared to be smaller in Co_3_O_4_/ZnO-25 than that in Co_3_O_4_/ZnO-15. Noteworthy, as the zinc acetate concentration increased further to 35.0 mM during fabrication of Co_3_O_4_/ZnO heterostructures, sunflower-seed-like crystals completely disappeared and the morphology of Co_3_O_4_/ZnO-35 represented hierarchical spheres (Fig. 6d). It was discovered that the inner part of Co_3_O_4_/ZnO sphere is assembled by the numerous nano-lamellas with thickness of 100–200 nm, as clearly indicated by the higher resolution SEM image in Fig. 7. The nano-lamellas are stacked together along the radial direction in interpenetrating network to form Co_3_O_4_/ZnO heterostructural spheres, which ultimately provided higher surface-to-volume ratio in this particular morphology. It is worthwhile to note that as zinc acetate concentration increased further to 45.0 mM, the sunflower-seed-like Co_3_O_4_/ZnO crystals reappeared again in smaller sizes and the new morphology of Co_3_O_4_/ZnO is established (Fig. 6e). In this morphology, Co_3_O_4_/ZnO nanorods have a diameter of approximately 700 nm. Thus, two kinds of crystal morphologies, sunflower-seed-like and nanorod crystals coexisted in Co_3_O_4_/ZnO-45 heterostructure. Finally, when the zinc acetate concentration reached 55.0 mM, the proportion and the size of Co_3_O_4_/ZnO rods increased significantly accompanied by their heavy agglomeration (Fig. 6f).

**Fig. 8:**
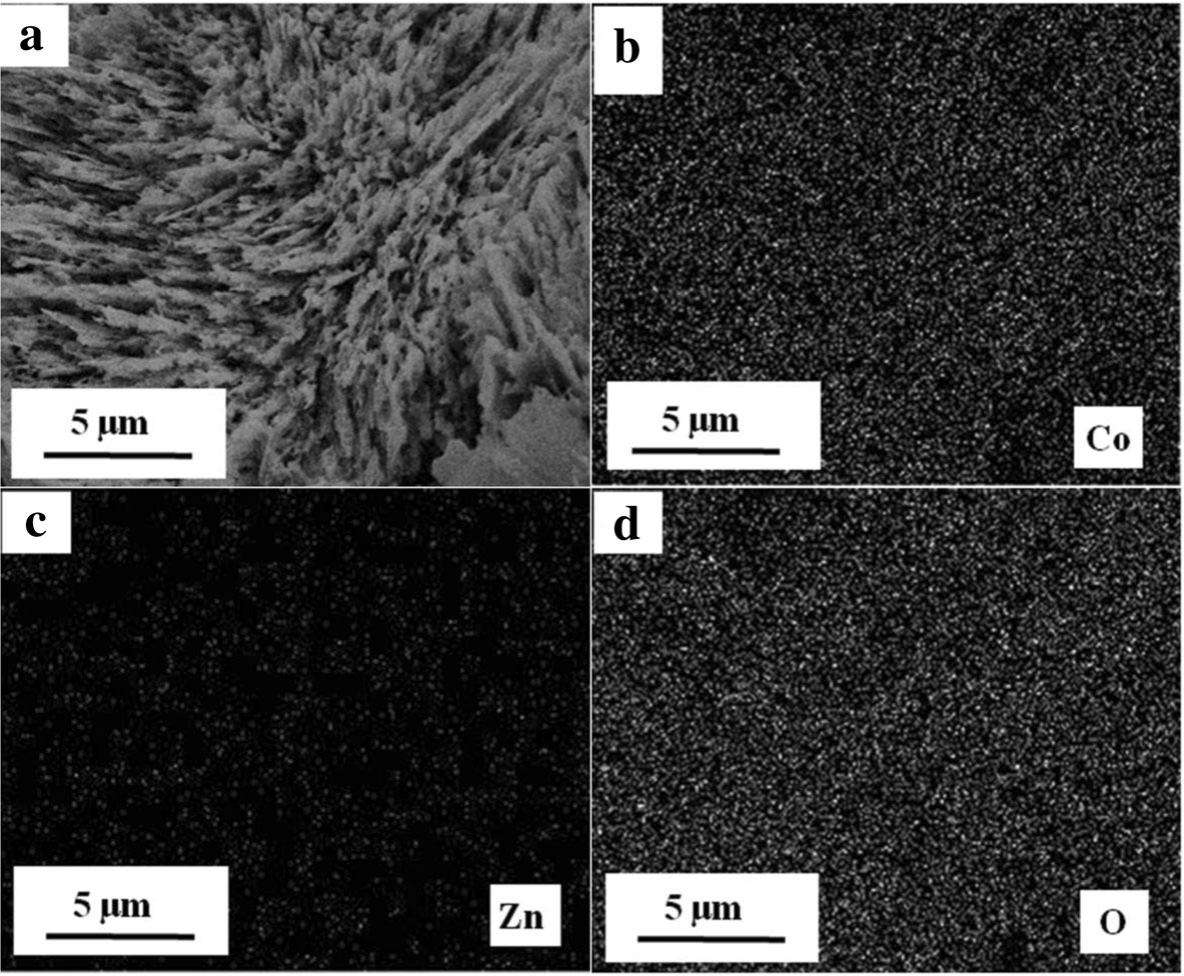
**a** SEM image of Co_3_O_4_/ZnO-35 composite with EDX mappings of **b** Co, **c** Zn, and **d** O

The elemental Zn, O, and Co in pure Co_3_O_4_ and Co_3_O_4_/ZnO heterostructures were detected by EDX, and the corresponding weight and atomic percentages for all samples are listed in Table 1. It is evident that the amount of zinc in Co_3_O_4_/ZnO heterostructures increases with the increasing concentration of zinc acetate. The elemental mappings of Co, Zn, and O in the Co_3_O_4_/ZnO-35 heterostructure are displayed in Fig. 8. It can be found that the Co, Zn, and O elements are concomitant and homogeneously distributed in the heterostructure.

**Table 1:** Weight and atomic percentages of elements in Co_3_O_4_ nanoparticles and Co_3_O_4_/ZnO heterostructures detected by EDX

Sample	Co_3_O_4_		Co_3_O_4_/ZnO-5			Co_3_O_4_/ZnO-15			Co_3_O_4_/ZnO-25			Co_3_O_4_/ZnO-35			Co_3_O_4_/ZnO-45			Co_3_O_4_/ZnO-55		
Element	Co	O	Zn	Co	O	Zn	Co	O	Zn	Co	O	Zn	Co	O	Zn	Co	O	Zn	Co	O
Wt.%	66.16	33.84	1.30	70.06	28.64	1.55	69.67	28.78	1.57	70.40	28.03	2.13	69.64	28.23	2.35	66.54	31.11	5.29	64.51	30.20
Atom%	34.67	65.33	0.66	39.64	59.70	0.79	39.34	59.87	0.81	40.21	58.98	1.09	39.67	59.24	1.16	36.31	62.53	2.64	35.74	61.62

Consequently, all the above material characterization techniques signify the successful and uniform development of the Co_3_O_4_/ZnO heterostructures. Thus, these *p*-Co_3_O_4_/*n*-ZnO heterostructures were formed without any impurity by the decomposition of Co(OH)_2_ and zinc acetate Zn(CH_3_COO)_2_ precursors calcined and annealed at 250 and 400 °C, respectively, by the following reactions [52, 53]:

1 $$ {\mathrm{Co}}^{2+}+6\ {\mathrm{NH}}_3\to {\left[\mathrm{Co}{\left({\mathrm{NH}}_3\right)}_6\right]}^{2+} $$

2 $$ 2\ {\left[\mathrm{Co}{\left({\mathrm{NH}}_3\right)}_6\right]}^{2+}+{\mathrm{O}}_2\ {\displaystyle \begin{array}{c}\mathrm{RT}\\ {}\rightleftharpoons \\ {}\Delta \end{array}}\ {\left[{\left({\mathrm{NH}}_3\right)}_5\mathrm{Co}{\mathrm{O}}_2{\left({\mathrm{NH}}_3\right)}_5\right]}^{4+}+2\ \mathrm{N}{\mathrm{H}}_3\uparrow $$

3 $$ {\left[\mathrm{Co}{\left({\mathrm{NH}}_3\right)}_6\right]}^{2+}+2\ {\mathrm{OH}}^{-}\ \overset{\Delta  }{\to }\ \mathrm{Co}{\left(\mathrm{OH}\right)}_2\downarrow +6\ \mathrm{N}{\mathrm{H}}_3\uparrow $$

4 $$ 6\ \mathrm{C}\mathrm{o}{\left(\mathrm{OH}\right)}_2+{\mathrm{O}}_2\ \overset{250{}^{\circ}\mathrm{C}}{\to }\ {\mathrm{Co}}_3{\mathrm{O}}_4+6{\mathrm{H}}_2\mathrm{O} $$

5 $$ \mathrm{Zn}{\left({\mathrm{CH}}_3\mathrm{COO}\right)}_2\bullet 2\ {\mathrm{H}}_2\mathrm{O}\ \overset{\Delta  }{\to }\ \mathrm{Zn}{\left({\mathrm{CH}}_3\mathrm{COO}\right)}_2+2\ {\mathrm{H}}_2\mathrm{O} $$

6 $$ \mathrm{Zn}{\left({\mathrm{CH}}_3\mathrm{COO}\right)}_2\ \overset{400{}^{\circ}\mathrm{C}}{\to }\ \mathrm{ZnO}+{\mathrm{CH}}_3\mathrm{COC}{\mathrm{H}}_3+{\mathrm{CO}}_2\uparrow $$

The BET surface areas of pure Co_3_O_4_ nanoparticles and Co_3_O_4_/ZnO heterostructures are presented in Table 2, and the corresponding nitrogen adsorption–desorption isotherms are depicted in Figure S1 of Additional file 1. With an increase of the zinc acetate concentration in the developed Co_3_O_4_/ZnO heterostructures, the BET surface area of samples initially increased and then decreased. For instance, the BET surface area of Co_3_O_4_/ZnO heterostructure reached to the largest level of 60.23 m^2^/g at the zinc acetate concentration of 35.0 mM. Larger surface area with more adsorption centers is more beneficial for the degradation of organic dyes [54].

**Table 2:** BET specific surface areas of pure Co_3_O_4_ nanoparticles and Co_3_O_4_/ZnO heterostructures

Sample	Co_3_O_4_	Co_3_O_4_/ZnO-5	Co_3_O_4_/ZnO-15	Co_3_O_4_/ZnO-25	Co_3_O_4_/ZnO-35	Co_3_O_4_/ZnO-45	Co_3_O_4_/ZnO-55
S_BET_/m^2^·g^−1^	23.97	38.51	40.14	43.48	60.23	26.63	24.19

**Fig. 9:**
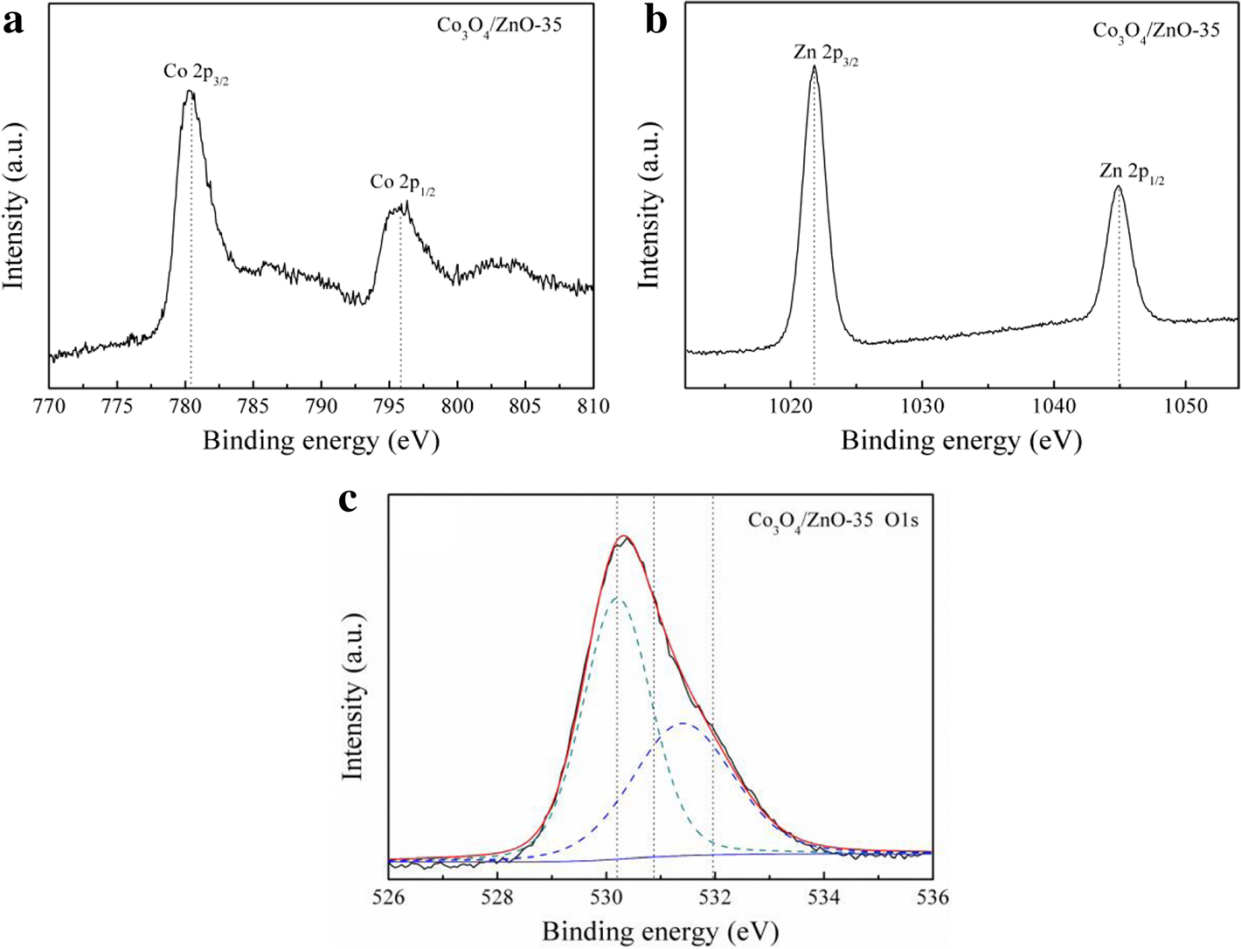
XPS spectra of **a** Co 2p, **b** Zn 2p, and **c** O 1s of *p*-Co_3_O_4_/*n*-ZnO heterostructure

To get further confirmation of the development *p*-Co_3_O_4_/*n*-ZnO heterostructures, part of the Co_3_O_4_/ZnO-35 structure was peeled off from the Ni substrate to perform XPS analysis. XPS measurements were performed to investigate the chemical binding states of the developed *p*-Co_3_O_4_/*n*-ZnO heterostructure. Figure 9 shows the results of XPS measurements, which were carried out to investigate the chemical binding states of the developed *p*-Co_3_O_4_/*n*-ZnO heterostructure. Figure 9a depicts the oxidation states of Co 2p in the XPS spectrum. Two main peaks Co 2p_3/2_ and Co 2p_1/2_ were clearly determined at 780.28 and 795.76 eV, respectively. Noteworthy, owing to complete coating of the porous Ni substrate, some noise level has been recorded at Co 2p_1/2_. In addition, the Zn 2p spectrum was also recorded during XPS measurements for *p*-Co_3_O_4_/*n*-ZnO heterostructure and this spectrum is presented in Fig. 9b. Two peaks for Zn 2p were also identified as Zn 2p_3/2_ and Zn 2p_1/2_ at binding energies of 1021.8 and 1044.9 eV, respectively. These results were in the line with other survey [39]. Figure 9c illustrates the O 1s regions for the *p*-Co_3_O_4_/*n*-ZnO heterostructure. Employing the Shirley background two deconvoluted Lorentzian-Gaussian peaks were obtained in O 1s spectrum. These peaks for *p*-Co_3_O_4_/*n*-ZnO heterostructure were clearly pronounced at 530.2 and 531.4 eV, respectively. The recorded peaks are comparable to the other peaks reported for lattice oxygen and chemisorbed oxygen of the surface hydroxyls [41, 55].

### Photocatalytic Activity

**Fig. 10:**
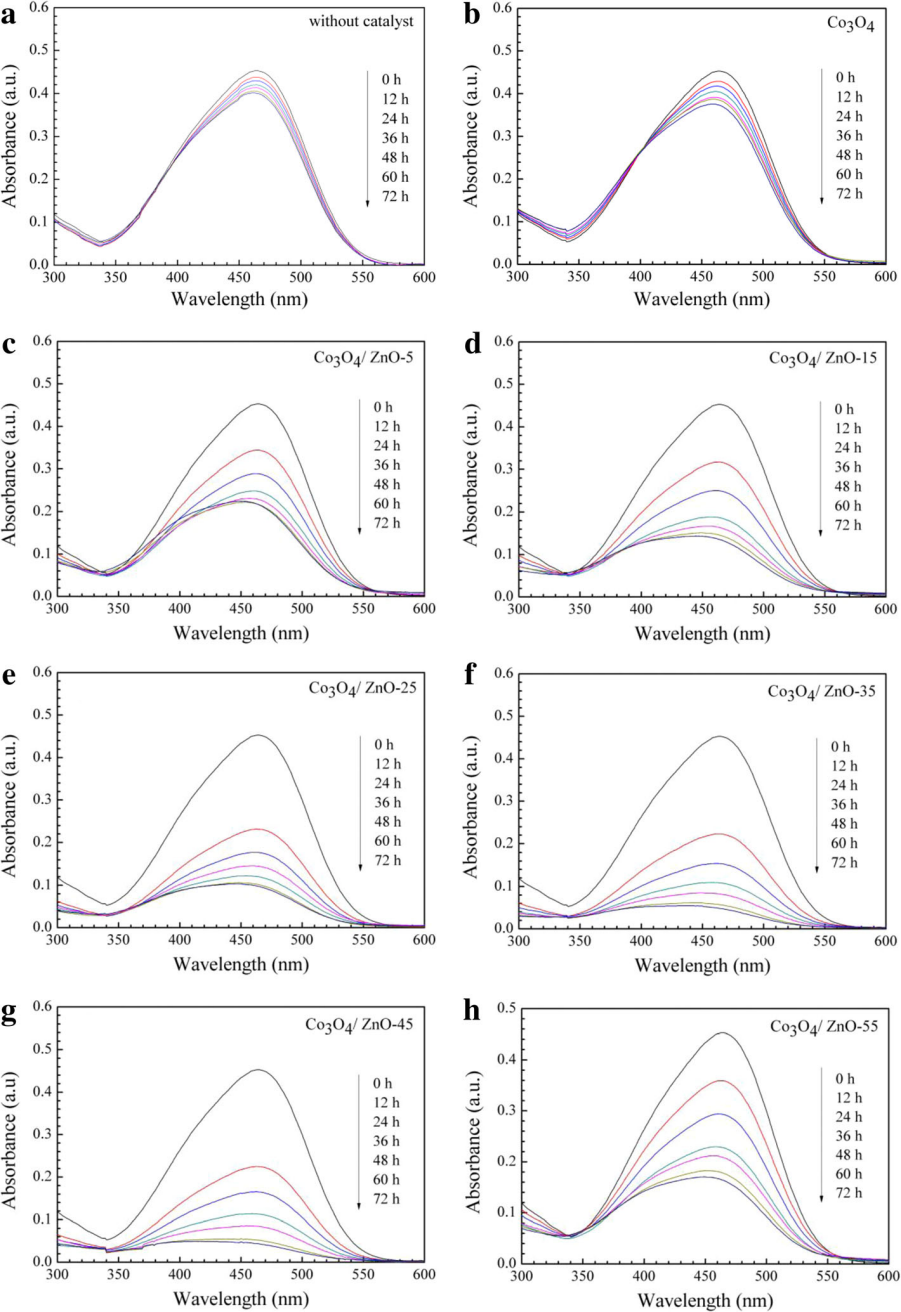
Irradiation time-dependent UV-vis absorbance spectra of MO aqueous solution: **a** without catalyst, and in the presence of **b** pure Co_3_O_4_, **c** Co_3_O_4_/ZnO-5, **d** Co_3_O_4_/ZnO-15, **e** Co_3_O_4_/ZnO-25, **f** Co_3_O_4_/ZnO-35, **g** Co_3_O_4_/ZnO-45, and **h** Co_3_O_4_/ZnO-55, respectively

**Fig. 11:**
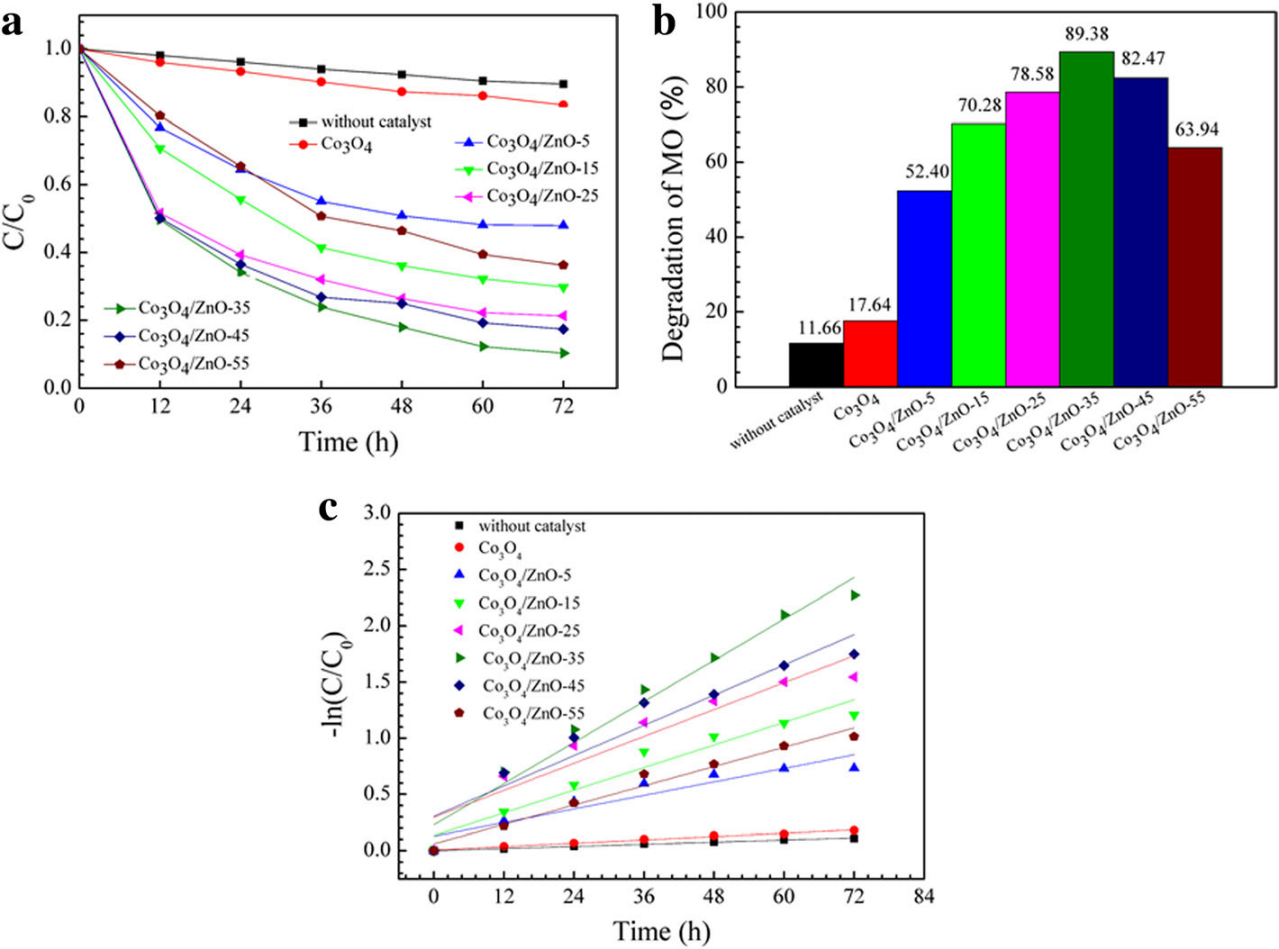
Photocatalytic activity of pure Co_3_O_4_ and the different fabricated *p*-Co_3_O_4_/*n*-ZnO heterostructures: **a** the relative concentration of MO as a function of irradiation time ((C_0_ and C are the concentration of MO at initial and any time)), **b** the degradation efficiency of MO in 72 h, **c** first-order plot of MO

The photocatalytic degradation of MO under the UV light irradiation (*λ* = 254 nm) was carried out at room temperature to evaluate the photocatalytic activity of the developed Co_3_O_4_ and *p*-Co_3_O_4_/*n*-ZnO heterostructures and specify the effect of zinc acetate concentration on the performance of *p*-Co_3_O_4_/*n*-ZnO heterostructures. The temporal spectral changes of MO aqueous solutions are displayed in Fig. 10. The corresponding relative concentration of MO with irradiation time and the performance of various *p*-Co_3_O_4_/*n*-ZnO heterostructures towards the MO degradation are presented in Fig. 11. As clearly visible from Fig. 10a, MO shown only negligible degradation with increasing irradiation time without catalysts and the degradation efficiency after 72 h of UV irradiation was only 11.66% (Fig. 11a). ZnO also shown poor photocatalytic activity (Figure S2 of Additional file 1). Pure Co_3_O_4_ demonstrated slightly better photocatalytic activity and the degradation efficiency was ~ 17.64% after 72 h irradiation (Fig. 11a). On the contrary, for the developed *p*-Co_3_O_4_/*n*-ZnO heterostructures utilized as catalysts, the main characteristic absorption peak (*λ* = 465 nm) of MO decreased with the increase of the irradiation time (Fig. 10c–h), which caused significant MO degradation. The first-order plot was fitted with this experiment, and the rate constant of MO degradation was obtained by the following equation

7 $$ \ln \left({\mathrm{C}}_0/{C}_t\right)= kt, $$

where *t* is the irradiation time, *C*_*0*_ is the initial concentration at time *t* = 0, *C*_*t*_ is the concentration at time *t*, and *k* is the first-order rate constant. As can be observed in Fig. 11c, the computed rate constants for Co_3_O_4_ and *p*-Co_3_O_4_/*n*-ZnO heterostructures are summarized in Table 3.

**Table 3:** First-order rate constants of pure Co_3_O_4_ nanoparticles and Co_3_O_4_/ZnO heterostructures

Sample	methyl orange	Co_3_O_4_	Co_3_O_4_/ZnO-5	Co_3_O_4_/ZnO-15	Co_3_O_4_/ZnO-25	Co_3_O_4_/ZnO-35	Co_3_O_4_/ZnO-45	Co_3_O_4_/ZnO-55
K (h^−1^)	0.00157	0.00245	0.01004	0.01697	0.01999	0.03054	0.02248	0.01432

**Fig. 12:**
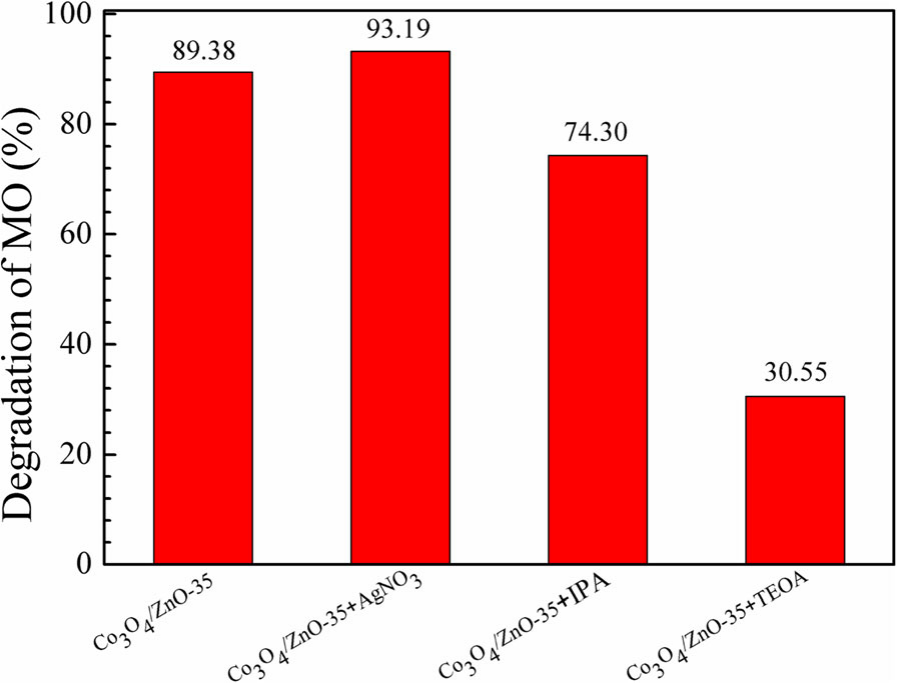
Trapping experiment of active species during the photocatalytic degradation of MO with Co_3_O_4_/ZnO-35 under 72 h UV light irradiation

The photocatalytic degradation of dyes mainly involves several active radical species such as hydroxyl radicals (·OH), holes (h^+^), and electrons (e^−^) [29]. In order to investigate the active species in the photocatalytic process to better understand the mechanism of photocatalysis, a series of scavengers were employed during the photo-degradation processes. Isopropanol (IPA), triethanolamine (TEOA), and silver nitrate (AgNO_3_) were used as scavengers for hydroxyl radicals (·OH), photo-generated holes, and electrons in degradation of MO, respectively [55–57]. The concentration of the three kinds of scavengers was 10 mM. Figure 12 shows the photocatalytic degradation of MO over Co_3_O_4_/ ZnO-35 heterostructure catalyst was 74.30, 30.55, and 90.25% with 10 mM IPA, TEOA, and AgNO_3_, respectively. This result means the photo-generated holes play much more important roles in MO degradation process, compared to ·OH and photo-generated electrons.

**Fig. 13:**
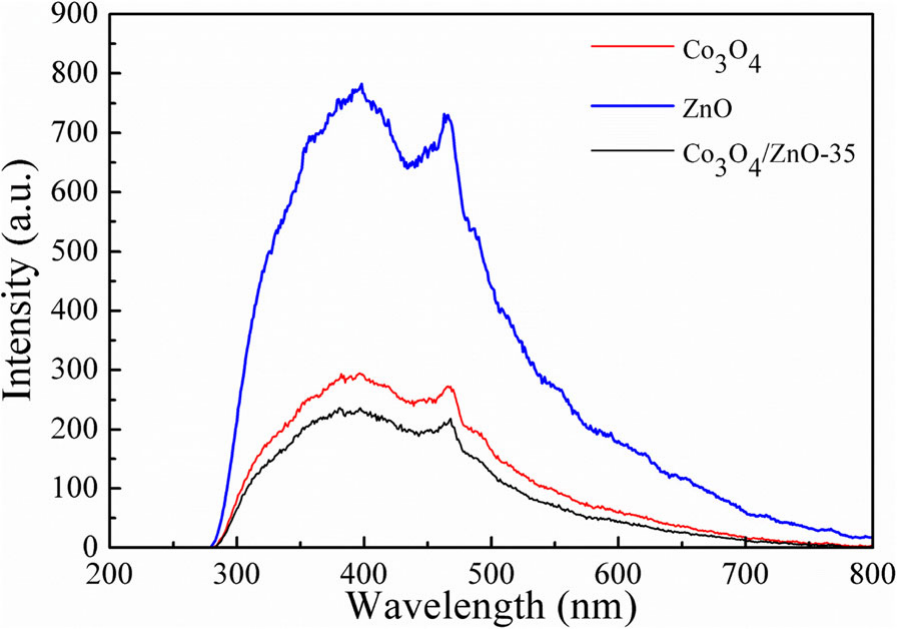
Room temperature PL spectra of the synthesized Co_3_O_4_, ZnO, and Co_3_O_4_/ZnO-35 heterostructure

Photoluminescence (PL) technique is widely used to investigate the recombination rate of the photo-induced electron-hole pairs in photocatalyst. Figure 13 shows the room temperature PL spectra of the synthesized Co_3_O_4_, ZnO, and Co_3_O_4_/ZnO-35 heterostructure (PL spectra of all samples are presented as Figure S3 in Additional file 1). There are two peaks in the PL spectra of Co_3_O_4_, ZnO, and Co_3_O_4_/ZnO heterostructures: one is called near band edge emission (NBE), which is in UV region and due to the recombination of free excitons through an exciton–exciton collision process; and the other one is called deep level emission (DPE, in visible region), which is caused by the impurities and structural defects in the crystal [58, 59]. The DPE intensity in Co_3_O_4_ and Co_3_O_4_/ZnO-35 heterostructures is much lower than in ZnO, which indicates that the recombination of the photo-generated charge carriers is harder in Co_3_O_4_ and Co_3_O_4_/ZnO-35 heterostructures than in ZnO. It has also been demonstrated that the recombination efficiency of photo-induced electron-hole pairs in ZnO can be effectively inhibited by the modification of In_2_O_3_ for the formation of heterojunction structure [56]. The DPE of Co_3_O_4_/ZnO-35 is little higher than pure Co_3_O_4_, and ZnO amount has no regular effect on the recombination rate of photo-generated charge carriers in Co_3_O_4_/ZnO heterostructures, which may be caused by the small quantity of ZnO and the increasing of defect concentration at the Co_3_O_4_/ZnO interface. This indicates that the composition of ZnO has little effect on the recombination of photo-generated electrons and holes in Co_3_O_4_.

**Fig. 14:**
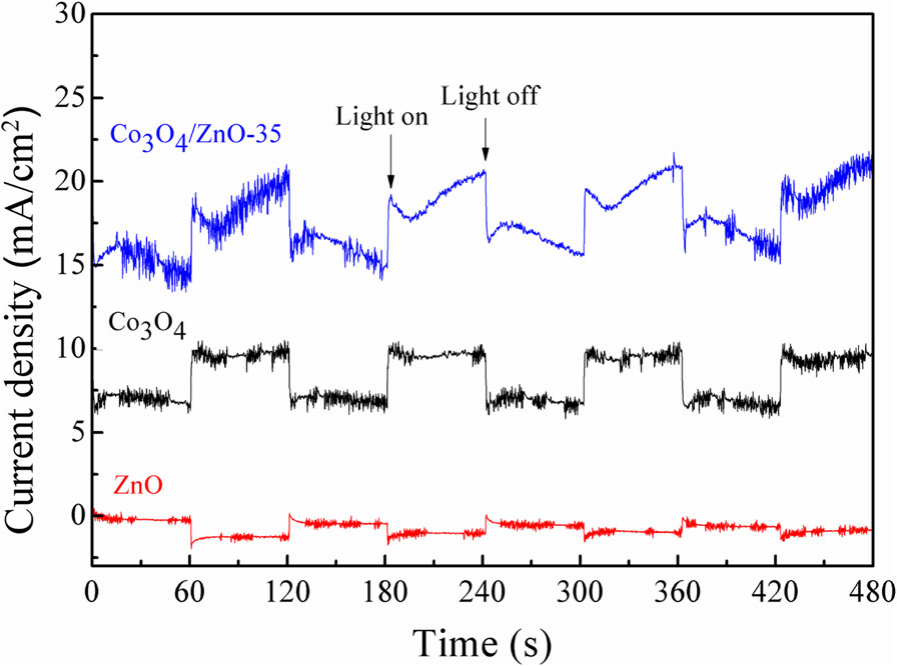
The photocurrent transient responses profiles for the synthesized Co_3_O_4_, ZnO and Co_3_O_4_/ZnO-35 heterostructure

For further investigation of photocatalytic activities of catalysts, the photocurrent transient responses of the synthesized Co_3_O_4_, ZnO, and Co_3_O_4_/ZnO heterostructures were measured under the visible light. Figure 14 depicts the photocurrent response of the synthesized Co_3_O_4_, ZnO, and Co_3_O_4_/ZnO-35 heterostructures. Notably, the photocurrent density of Co_3_O_4_/ZnO-35 is much higher than that of ZnO and Co_3_O_4_, which indicated that the Co_3_O_4_/ZnO-35 has the highest photocatalytic activity [29].

**Fig. 15:**
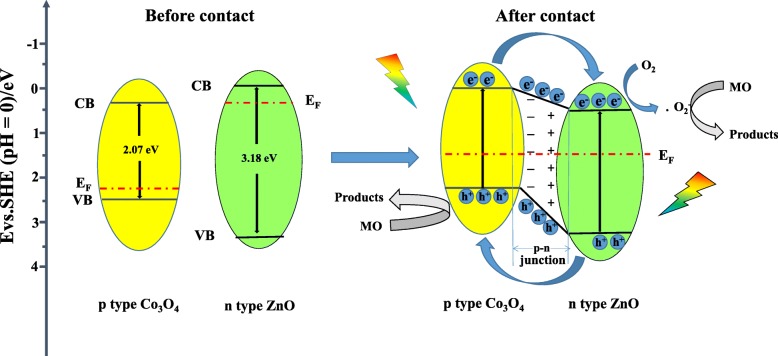
Schematic diagram of photocatalytic mechanism of Co_3_O_4_/ZnO-35 composites under UV irradiation

According to the results above, the improvement of photocatalytic activity of Co_3_O_4_ by additional incorporation of ZnO is mainly caused by two ways. The first one is based on the fact that the increasing concentration of photo-generated holes in Co_3_O_4_ accelerates the photocatalytic rate. As illustrated in Fig. 15, the valence bands (VB) of Co_3_O_4_ and ZnO are 2.44 V/SHE [60] and 3.03 V/SHE [56], respectively. And the conduction bands (CB) of Co_3_O_4_ and ZnO are 0.37 V/SHE [60] and − 0.15 V/SHE [56], respectively. After incorporation of *n*-type ZnO the with *p*-type Co_3_O_4_, the energy levels of Co_3_O_4_ shift upward, whereas the energy band of ZnO shifts downward until the Fermi energy (E_F_) of Co_3_O_4_ and ZnO reaches an equilibrium. The newly formed energy band structure became to the interactive structure [61]. A large number of *n*-type ZnO nanoparticles are tightly assembled on the surface of *p*-type Co_3_O_4_. Thus, a large number of nano *p-n* junctions are formed on the surface of Co_3_O_4_. Under irradiation, both Co_3_O_4_ and ZnO absorb light and the excited electrons migrate to the CBs whereas the holes remain on the VB of both Co_3_O_4_ and ZnO. The electrons on the CB of Co_3_O_4_ could easily transfer to the CB of ZnO. Simultaneously, the holes in the VB of ZnO migrate into the VB of Co_3_O_4_; thus, the concentration of photo-generated holes on Co_3_O_4_ surface increases. Based on the data presented in Fig. 12, photo-generated holes play the most important role in photo-degradation process of MO on *p*-Co_3_O_4_/*n*-ZnO heterostructures. Thus, the increasing concentration of photo-generated holes in the Co_3_O_4_ VB could lead to its highest photocatalytic activity.

The second way of improvement of photo-catalytic activity is caused by the high-specific surface area of the *p*-Co_3_O_4_/*n*-ZnO heterostructures. The molecules’ absorption-desorption on the surface of catalyst is the first step in degradation process [54, 62]. Consequently, higher surface-to-volume ratio in the developed morphology of *p*-Co_3_O_4_/*n*-ZnO heterostructures provides more unsaturated surface coordination sites, as shown in Table 2. The *p*-Co_3_O_4_/*n*-ZnO heterostructures possess higher specific surface area caused by numerous ultrathin nano-lamellas, as confirmed by SEM characterizations. Therefore, high surface-to-volume ratio and suitable interfaces obtained for the Co_3_O_4_/ZnO-35 heterostructure resulted in its outstanding photocatalytic activity towards the efficient MO degradation.

It needs to note that with the zinc acetate concentration increasing higher than 35.0 mM, the photocatalytic activities of Co_3_O_4_/ZnO heterostucture decreases. This could be caused by the decrease of their specific surface area (as presented in Table. 2). The similar trend was also observed for the tetracycline (TC) degradation by Mn-doped SrTiO_3_ nanotubes with the increase of Mn dopant concentration [63]. Thus, with certain increase of the zinc acetate concentration, the quantity of ZnO increases and the mass of electron-hole pairs within the space charge region is efficiently separated by the Co_3_O_4_/ZnO interface resulting in the improvement of MO degradation efficiency.

**Fig. 16:**
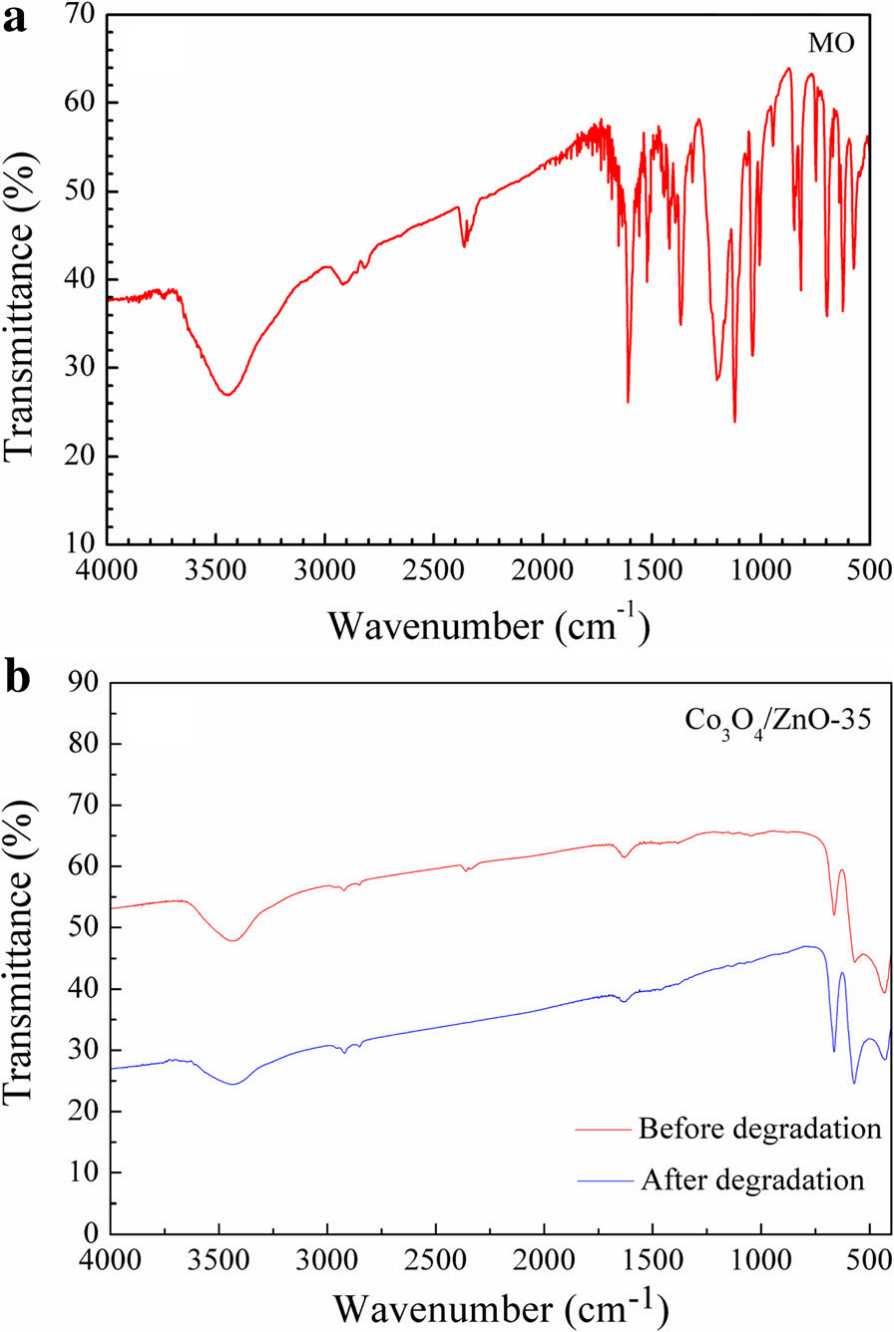
FTIR spectra of **a** MO, **b** Co_3_O_4_/ZnO-35 heterostructure after 72 h degradation of MO

Noteworthy, the color of both Co_3_O_4_ and *p*-Co_3_O_4_/*n*-ZnO heterostructures is not varied from their original one after ~ 72 h of the MO degradation, whereas the color of MO-containing solution faded away from the initial lemon’s yellow to almost transparent and even diminished with the Co_3_O_4_/ZnO-35 heterostructure. FTIR spectrum of Co_3_O_4_/ZnO-35 heterostructure after 72 h degradation of MO were shown in Fig. 16 (FTIR spectra of all samples are presented in Figure S4 of Additional file 1). No MO adsorption peak appeared in the FTIR spectrum of Co_3_O_4_/ZnO-35 heterostructure immersed 72 h in MO solution, indicating that MO molecules are degraded to the smaller molecules [64, 65].

All the above experiments relevant to investigation of the photocatalytic activity of fabricated *p*-Co_3_O_4_/*n*-ZnO heterostructures undoubtedly confirmed that the MO degradation under the UV light illumination is relatively slow without the presence of catalyst. The presence of *p*-Co_3_O_4_/*n*-ZnO heterostructures as catalysts significantly increased the rate of MO degradation under the same UV light irradiation conditions. The 35 mM of zinc acetate concentration used in preparation of *p*-Co_3_O_4_/*n*-ZnO heterostructures has provided the essential prerequisite for development of unique and well-structured morphology with high-surface-to-volume ratio, which subsequently resulted in the maximum photocatalytic activity of the *p*-Co_3_O_4_/*n*-ZnO heterostructure for the MO degradation. These experimental results indirectly confirmed the fact that the catalytic process was mainly related to the adsorption and desorption of molecules on the large surface area of catalysts. High-surface-to-volume ratio provided more unsaturated surface coordination sites, which in turn endowed *p-n* heterojunction with enhanced photocatalytic activity [66].

## Conclusions

Different *p*-Co_3_O_4_/*n*-ZnO heterostructures were successfully fabricated by the hydrothermal decomposition method on the porous Ni substrate with the different zinc acetate concentration varying from 5.0 to 55.0 mM as a ZnO source. The resulted *p*-Co_3_O_4_/*n*-ZnO heterostructures illustrated various structural morphologies. The synthesized *p*-Co_3_O_4_/*n*-ZnO heterostructures were subjected to the water treatment as photocatalysts under the UV light irradiation. The reaction rate of MO degradation at the room temperature and at the presence of these photocatalysts was substantially promoted. In fact, *p*-Co_3_O_4_/*n*-ZnO heterostructures exhibited much higher photocatalytic activity than that of pure Co_3_O_4_ for MO degradation. It was discovered that the photocatalytic activity of *p*-Co_3_O_4_/*n*-ZnO heterostructures is greatly affected by the zinc acetate concentration. The optimum zinc acetate concentration was found to be at 35%. At this concentration, the synthesized Co_3_O_4_/ZnO displayed unique hierarchical nano-lamellar sphere structure and also demonstrated the highest photocatalytic activity among other samples with the different zinc acetate concentration. Co_3_O_4_/ZnO-35 reached the degradation efficiency of 89.38% for MO decomposition in 72 h of irradiation. With further increase of the zinc acetate concentration, the resulted *p*-Co_3_O_4_/*n*-ZnO heterostructures demonstrated lower photocatalytic activity towards MO degradation at room temperature. In contrast to the pure Co_3_O_4_ component, the fabricated *p*-Co_3_O_4_/*n*-ZnO heterostructures possess higher concentration of photo-generated holes and larger specific surface area, which leads to its enhanced photocatalytic activity.

## Methods

### Materials Synthesis

All solvents and reagents were purchased from the commercial sources and represented analytical grade. They were used and received without further purification. *p*-Co_3_O_4_/*n*-ZnO heterostructures were prepared by two-step fabrication method on the porous Ni substrate (25 mm × 25 mm × 1 mm). Initially, the Ni substrates were thoroughly cleaned by acetone and deionized water at the room temperature. Then, they were immersed into 6 M hydrochloric acid and 0.1 M nickel chloride solution for 10 min. After that treatment, the cleaned Ni substrates were dried for further use.

For fabrication of *p*-Co_3_O_4_/*n*-ZnO heterostructures on the Ni substrates, 1.7463 g Co(NO_3_)_2_·6H_2_O was firstly dissolved in 18 mL of deionized water and stirred for approximately 5 min until the solution turned pink and gradually turned into black by the addition of 12 mL 28 wt.% ammonia solution. pH of solution was 12. Then, both the solution and cleaned Ni substrate were transferred into 50 mL Teflon-lined stainless steel autoclave with subsequent heat-treatment at 120 °C for 10 h. Upon completion of the reaction, the autoclave was cooled to the room temperature and the pH of solution becomes 10.7. The Ni substrate with pink precursor was taken out, washed, and dried with the following calcination at 250 °C for 1 h in ambient air to get Co_3_O_4_ particles.

At the second step, the Ni substrates with the developed Co_3_O_4_ particles were immersed for 2 h into zinc acetate Zn(CH_3_COO)_2_ dispersed in N,N–dimethylformamide (DMF) solutions with the different zinc acetate concentrations of 5.0, 15.0, 25.0, 35.0, 45.0, and 55.0 mM, respectively. After that step, the Ni substrates with loaded Co_3_O_4_/ Zn(CH_3_COO)_2_ structures were dried in air at the room temperature. Finally, they were annealed in the tube furnace at 400 °C for 30 min, the heating rate of 5 °C/min to develop *p*-Co_3_O_4_/*n*-ZnO heterostructures. The fabricated *p*-Co_3_O_4_/*n*-ZnO heterostructures obtained at the different zinc acetate concentrations were labeled as Co_3_O_4_/ZnO-5, Co_3_O_4_/ZnO-15, Co_3_O_4_/ZnO-25, Co_3_O_4_/ZnO-35, Co_3_O_4_/ZnO-45, and Co_3_O_4_/ZnO-55, respectively.

### Characterization

The crystal structure of precursor, Co_3_O_4_ particles, and *p*-Co_3_O_4_/*n*-ZnO heterostructures fabricated on the Ni substrates were characterized by D/Max-rB X-ray diffractometer (XRD) with a Cu-K_α1_ radiation (*λ* = 0.1542 nm) operating at 100 mA and 40 kV and a scan rate of 5°/min. Scanning electron microscopy (SEM) and energy dispersive X-ray (EDX) spectroscopy were carried out by a SU-5000 microscope equipped with EDX attachment. The Raman spectra were recorded on a Renishaw in Via Raman microscope, and a 514.5-nm Ar^+^ laser line with a power output of 20 mW was used for excitation with a spectral resolution of 2 cm^− 1^. Fourier Transform Infrared (FTIR) spectra were taken using a NEXUS Thermo Nicolet IR-spectrometer in the range 4000–500 cm^− 1^ with a spectral resolution of 2 cm^− 1^ by KBr disk method. X-ray photoelectron spectroscopy (XPS) was employed in order to investigate the surface chemistries of the developed samples in ESCALAB system with AlK X-ray radiation at 15 kV. All XPS spectra were accurately calibrated by the C 1s peak at 284.6 eV to compensation of the charge effect. Brunauer-Emmett-Teller (BET, JW-BK122F, China) was applied to analyze the specific surface area. Room temperature photoluminescence (PL) spectra of the synthesized Co_3_O_4_ and *p*-Co_3_O_4_/*n*-ZnO heterostructures were performed on an F-4600 fluorescent spectrophotometer (Hitachi Corp., Tokyo, Japan), the maximal excitation wavelength was 200 nm, and the filter was 300 nm.

### Photocatalytic Activity Evaluation

The photocatalytic activity of both as-fabricated Co_3_O_4_ and *p*-Co_3_O_4_/*n*-ZnO heterostructures developed on the Ni substrates for the MO (C_14_H_14_N_3_NaO_3_S) degradation in aqueous solution under the UV light was evaluated by measuring absorbance of the irradiated solution. For this study, Ni substrates attached with the different *p*-Co_3_O_4_/*n*-ZnO heterostructures were placed into 100 mL of MO solutions with a concentration of 6 mg/L and pH of 6.5. The solutions were continuously stirred in dark for 2 h before illumination in order to reach the absorption-desorption equilibrium between MO and the *p*-Co_3_O_4_/*n*-ZnO heterostructures. Then, the solutions were irradiated by 30 W low-pressure UV lamp (*λ* = 254 nm), which was located at the distance of 50 cm above the top of the dye solution. During the process, 5 mL solutions were pipetted every 12 h for the absorbance determination by a UNIC UV-2800A spectrophotometer using the maximum absorbance at 465 nm. All experiments were performed under the ambient condition and room temperature. The degradation efficiency of MO was defined as:

8 $$ D=\left[\left({\mathrm{A}}_0-{A}_t\right)/{A}_0\right]\times 100\%, $$

where *D* is degradation efficiency, *A*_*0*_ is the initial absorbance of MO solution, and *A*_*t*_ is the absorbance of MO solution after UV irradiation within the elapsed time *t*.

### Photo-Electrochemical Characterization

The photocurrent measurements were carried out at an open circuit potential using an electrochemical workstation (CHI-660e, Chenhua Instrument Corp., China). A three-electrode system was used with the prepared Co_3_O_4_ or Co_3_O_4_/ZnO samples, Pt plate, and saturated calomel electrode (SCE) acted as working, counter, and reference electrodes, respectively. A 300 W Xe lamp with an optical filter (AM 1.5 G) was employed as the excitation light source and NaOH solution (1 M) was used as the electrolyte.

## Additional File

Additional file 1: **Figure S1.** Nitrogen adsorption–desorption isotherms of (a) Co3O4, (b) Co3O4/ZnO-5, (c) Co3O4/ZnO-15, (d) Co3O4/ZnO-25, (e) Co3O4/ZnO-35, (f) Co3O4/ZnO-45, and (g) Co3O4/ZnO-55. Figure S2. Irradiation time dependent UV-vis absorbance spectra of MO aqueous solution in the presence ZnO. Figure S3. PL spectra of (a) ZnO, Co3O4, and Co3O4/ZnO heterostructures, and (b) the magnification of the square in (a). Figure S4. FTIR spectra of Co3O4 and Co3O4/ZnO heterostructures after 72 h degradation of MO. (DOCX 779 kb)

## Abbreviations

DMF: N,N–dimethylformamide
EDX: Energy dispersive X-ray
FTIR: Fourier transform infrared
MO: Methyl orange
SEM: Scanning electron microscopy
UV–vis: Ultraviolet-visible
XPS: X-ray photoelectron spectroscopy
XRD: X-ray diffraction
