# Supplementary material for: Effect of Zinc Acetate Concentration on Optimization of Photocatalytic Activity of p-Co3O4/n-ZnO Heterostructures
Source: Nanoscale Res Lett. 2018 Jul 5;13:195. doi: 10.1186/s11671-018-2604-4 (PMC6033848; doi:10.1186/s11671-018-2604-4)
Supplement: Supplementary file 1 — Figure S1. Nitrogen adsorption–desorption isotherms of (a) Co3O4, (b) Co3O4/ZnO-5, (c) Co3O4/ZnO-15, (d) Co3O4/ZnO-25, (e) Co3O4/ZnO-35, (f) Co3O4/ZnO-45, and (g) Co3O4/ZnO-55. Figure S2. Irradiation time dependent UV-vis absorbance spectra of MO aqueous solution in the presence ZnO. Figure S3. PL spectra of (a) ZnO, Co3O4, and Co3O4/ZnO heterostructures, and (b) the magnification of the square in (a). Figure S4. FTIR spectra of Co3O4 and Co3O4/ZnO heterostructures after 72 h degradation of MO. (DOCX 779 kb) [file 11671_2018_2604_MOESM1_ESM.docx]

Additional file 1

**Effect of Zinc Acetate Concentration on Optimization of Photocatalytic Activity of *p-*Co_3_O_4_/*n*-ZnO Heterostructures**

Hongyan Xu^a^, Mingliang Shi^a^, Caiqin Liang^a^, Siyan Wang^a^, Chengkai Xia^a^, Chenyang Xue^b^, Zhenyin Hai^c^, Serge Zhuiykov^*a,c^

^a^ School of Materials Science and Engineering, North University of China, Taiyuan 030051, P.R. China

^b^ Key Laboratory of Instrumentation Science and Dynamic Measurement of Ministry of Education, North University of China, Taiyuan 030051, P.R. China

^c^ Ghent University Global Campus, Department of Applied Analytical & Physical Chemistry, 119 Songdomunhwa-ro, Yeonsu-gu, Incheon 21985, South Korea

* - corresponding author; e-mail: serge.zhuiykov@ugent.be (S. Zhuiykov)

















Figure S1. Nitrogen adsorption–desorption isotherms of (a) Co_3_O_4_, (b) Co_3_O_4_/ZnO-5, (c) Co_3_O_4_/ZnO-15, (d) Co_3_O_4_/ZnO-25, (e) Co_3_O_4_/ZnO-35, (f) Co_3_O_4_/ZnO-45 and (g) Co_3_O_4_/ZnO-55.





Figure S2. Irradiation time dependent UV-vis absorbance spectra of MO aqueous solution in the presence ZnO.




Figure S3. PL spectra of (a) ZnO, Co_3_O_4_ and Co_3_O_4_/ZnO heterostructures, and (b) the magnification of the square in (a).





Figure S4. FTIR spectra of Co_3_O_4_ and Co_3_O_4_/ZnO heterostructures after 72 h degradation of MO.
